# FROM INCIPIENT TO SUBSTANTIAL: EVOLUTION OF PLACENTOTROPHY IN A PHYLUM OF AQUATIC COLONIAL INVERTEBRATES

**DOI:** 10.1111/evo.12039

**Published:** 2013-02-04

**Authors:** Andrew N Ostrovsky, D Fairbairn

**Affiliations:** 1 Department of Palaeontology, Faculty of Earth Sciences, Geography and Astronomy, Geozentrum, University of ViennaAlthanstrasse 14, A-1090, Vienna, Austria; 2 Department of Invertebrate Zoology, Faculty of Biology and Soil Science, St. Petersburg State UniversityUniversitetskaja nab. 7/9, 199034, St. Petersburg, Russia

**Keywords:** Brooding, Bryozoa, Cheilostomata, matrotrophy, oogenesis

## Abstract

Matrotrophy has long been known in invertebrates, but it is still poorly understood and has never been reviewed. A striking example of matrotrophy (namely, placentotrophy) is provided by the Bryozoa, a medium-sized phylum of the aquatic colonial filter feeders. Here I report on an extensive anatomical study of placental analogues in 21 species of the bryozoan order Cheilostomata, offering the first review on matrotrophy among aquatic invertebrates. The first anatomical description of incipient placentotrophy in invertebrates is presented together with the evidence for multiple independent origins of placental analogues in this order. The combinations of contrasting oocytic types (macrolecithal or microlecithal) and various degrees of placental development and embryonic enlargement during incubation, found in different bryozoan species, are suggestive of a transitional series from the incipient to the substantial placentotrophy accompanied by an inverse change in oogenesis, a situation reminiscent of some vertebrates. It seems that matrotrophy could trigger the evolution of sexual zooidal polymorphism in some clades. The results of this study show that this phylum, with its wide variety of reproductive patterns, incubation devices, and types of the simple placenta-like systems, offers a promising model for studying parallel evolution of placentotrophy in particular, and matrotrophy in general.

Key evolutionary novelties are often considered as a driving force of progressive evolution. Appearing at the genetic level and then affecting development, such innovations trigger a cascade of changes in various aspects of morphology, physiology, and reproduction of the animals (for reviews, see Nitecki 1990; Müller and Wagner 1937; Heard and Houser 1996; Wagner and Lynch 1940).

A celebrated example of an evolutionary novelty is matrotrophy or extraembryonic nutrition (EEN), the direct postfertilization provisioning of an embryo by its parent during incubation (Wourms 1975; Blackburn 1992; Marsh-Matthews 1998). This highly effective mode of parental care evolved independently in more than half of all the metazoan phyla. Matrotrophy is known in sponges, cnidarians, flat and round worms (both free-living and parasitic), nemerteans, annelids, molluscs, bryozoans, entoprocts, arthropods, onychophorans, echinoderms, chordates, and some other groups (see Giese and Pearse 1974, 1975a, 1975b, 1977; Giese et al. 1979, 1991; Adiyodi and Adiyodi [Bibr b1], [Bibr b2]; Blackburn 2005; Wourms et al. 1988, and references therein). The simplest mode of matrotrophy is histotrophy (absorption via embryonic surface cells), whereas the most complex mode is placentotrophy that according to Mossman's (2011, p. 156) widely accepted definition of placenta involves “any intimate apposition or fusion of the fetal organs to the maternal [or paternal] tissues for physiological exchange.” In addition to the therian mammals, placentas are widely documented among squamate reptiles, fishes (reviewed in Wourms 1975; Wourms et al. 1988; Wourms and Lombardi 1969; Blackburn 1992, 1993, 199a, 2005; Blackburn et al. 2006; Wooding and Burton 2008) and invertebrates, being often referred to as placental analogues or, sometimes, pseudoplacentas (Turner 2009; Hagan 1951; Blackburn 1992; Farley 1982). Despite the two latter terms being considered archaic, the “placental analogue” still seems suitable for describing simplest placentas of some invertebrates. The main reason for this is that close apposition between the embryo and the nutritive organ or tissue is often established during the later stages of incubation. Prior to this, the embryo absorbs nutrients from the surrounding fluid of the incubation chamber without any contact with the maternal wall (that provides nutrition).

Most of the phyla that feature EEN contain just a few matrotrophic species. However, there are some taxonomic groups where all the species possess EEN. Such groups are the platyhelminth class Trematoda, the arthropod orders Scorpiones, Pseudoscorpiones and Strepsiptera, the chordate order Salpida, and the class Mammalia (Hagan 1951; Weygoldt 1913; Francke 2003; Godeaux 1982; Lombardi 1995; Galaktionov and Dobrovolskij 2009). Among these taxa, only salps, nonmonotreme mammals, and scorpions possess placentas or placenta-like organs. Another striking example is the phylum Bryozoa, a medium-sized group of aquatic colonial filter-feeders, which is unique among invertebrates in possessing matrotrophy in all its major classes: all the living representatives of the classes Stenolaemata and Phylactolaemata and many species from the class Gymnolaemata are known to be matrotrophic (for review and history of the research see Reed 2011; Ostrovsky et al. 2007, see also Supplementary Materials 1). Moreover, it seems that most, if not all, bryozoans with EEN are placentotrophic.

Despite the rather broad distribution of matrotrophy in invertebrates, this phenomenon has never been reviewed in any invertebrate group except insects (see Hagan 1951; Meier et al. 2010). This article, presenting anatomical descriptions of the recently discovered placental analogues (embryophores) in 21 species of the bryozoan order Cheilostomata (Gymnolaemata), constitutes the first review on matrotrophy among aquatic invertebrates. EEN in these species is evidenced by (1) increase of the embryophore cells in number and their hypertrophy; (2) change in their cytological characteristics (deep staining of the cytoplasm and appearance of granules and/or vacuoles); (3) the enlargement of the embryo; (4) change in the size, shape and distribution pattern of the yolk granules in embryos; and (5) strong difference between the egg and incubation cavity size. The discovery of different degrees of embryonic enlargement and of morphological development of placental analogues (from small to well developed with modest and strong hypertrophy of the embryophore cells) in combination with the different modes of oogenesis (microlecithal or macrolecithal) resulted in reconstruction of the stages in the evolution of placentotrophy in this group. The understanding of reproductive patterns employing the simplest placenta-like systems in invertebrates may be important for reconstructing the evolution of matrotrophy in general.

## Materials and Methods

Specimens were collected by various methods (see Supplementary Materials 2) and fixed either in 2.5% glutaraldehyde (buffered in 0.1M Na-cacodylate with 10.26% sucrose, pH 7.3) or Bouin's fluid without acetic acid. While still in the fixative, the specimens were decalcified for 6 to 12 hours using a few drops of a 2N solution of hydrochloric acid, gradually dehydrated in an ethanol series, embedded in epoxy resin type TAAB 812, sectioned (1.0-μm thick), and stained with Richardson's stain using standard methods.

Placental analogues have been found and described in 21 species belonging to 18 genera of 12 families of Cheilostomata (Tables 1 and 2). The diameters of mature (ovarian and/or ovulated) oocytes, zygotes, and embryos were measured, and their volume calculated to compare their enlargement during incubation. Seasonal and geographic influence on reproduction cannot be considered, as every species was collected only once in one location.

**Table 1:** Maximum size of mature oocytes (or recently oviposed zygotes) and early/late embryos, and embryonic enlargement during incubation in the cheilostome species with pattern III

Species (Family)	Mature Oocyte (Zygote) (μm)	Early/Late Embryo (μm)	Embryonic Enlargement
*Bugula flabellata*	96.0 × 55.0	—/160.0 × 120.0	6.3-fold
(Bugulidae)	(77.0 μm in Dyrynda and King 1982)	(150.0 μm in Dyrynda and King 1982)	(7.1 in Dyrynda and King 1982)
	(80. 0 μm in Corrêa 1991)	(130. 0 μm in Corrêa 1991)	
*Bugula neritina*	32.0 × 30.0 (submature)	—/230.0 × 190.0	310-fold
(Bugulidae)	(zygote 36.0 μm in Woollacott and Zimmer 1972)	(larva 200.0–300.0 × 300.0–400.0 μm in Woollacott and Zimmer 1972)	(∼500 in Woollacott and Zimmer 1972)
*Cellaria fistulosa*	90.0 × 57.5	75.0 × 70.0/127.0 × 125.0	4.9-fold
(Cellariidae)			
*Mollia multijuncta*	33.6 × 28.8	55.0 × 45.0/175.0 × 60.0	53.41-fold
(Microporidae)			
*Pterocella scutella*	63.0 × 62.5	235.0 × 170.0/—	>33-fold
(Catenicellidae)			
*Urceolipora nana*	100.0 × 50.0	—/180.0 × 145.0	>10.17-fold
(Urceoliporidae)			
*Reciprocus regalis*	54.0 × 45.0	—/370.0 × 260.0	257.7-fold
(Urceoliporidae)			
*Gregarinidra serrata* (Flustridae)^1^	100.0 × 75.0	102.0 × 75.0/––	?

1 **M**entioned as having reproductive pattern II in Ostrovsky et al. (2007) by mistake.

**Table 2:** Maximum size of mature oocytes (or recently oviposed zygotes) and early/late embryos, and embryonic enlargement during incubation in the cheilostome species with pattern IV

Species (Family)	Mature Oocyte (Zygote) (μm)	Early/Late Embryo (μm)	Embryonic Enlargement
*Klugeflustra antarctica*	—	260.0 × 200.0 (smallest)/—	>1.5-fold
(Flustridae)^1^		310.0 × 220.0 (largest)/—	
*Isosecuriflustra angusta*	180.0 × 170.0	—/225.0 × 155.0	1.27-fold
(Flustridae)			
*Beania bilaminata*	55.2 × 50.4	115.0 × 85.0/490.0 × 330.0	468.2-fold
(Beaniidae)			
*Micropora notialis*	105.0 × 90.0	210.0 × 135.0/—	>1.5-fold
(Microporidae)			
*Cellaria tenuirostris*	85.0 × 58.0	90.0 × 77.0/115.0 × 100.0	3.39-fold
(Cellariidae)			
*Figularia figularis*	240.0 × 180.0	305.0 × 180.0/260.0 × 220.0	1.49-fold
(Cribrilinidae)			
*Cribricellina cribraria*	370.0 × 300.0	—/560.0 × 440.0	3.3-fold
(Catenicellidae)			
*Costaticella solida*	—	190.0 × 165.0/365.0 × 240.0	>4.9-fold
(Catenicellidae)^1^			
*Costaticella bicuspis*	—	320.0 × 210.0 (smallest)/—	>2.1-fold
(Catenicellidae)^1^		420.0 × 260.0 (largest)/—	
*Celleporella hyalina*	80.0 × 70.0	170.0 × 100.0/170.0 × 140.0	8.8-fold
(Hippothoidae)	(80.0 μm in Hughes 1995)	(larva ∼200.0 μm in Hughes 1995)	(15.6 in Hughes 1995)
		90.0/185.0–200.0	
		(in Cancino and Hughes 1999)	
“*Calyptotheca” variolosa* (Lanceoporidae)	250.0 × 190.0	260.0 × 180.0/460.0 × 320.0	5.57-fold
*Watersipora subtorquata* (Watersiporidae)^1^	—	126.2 × 100.0/163.0 × 147.5	3-fold
*Myriapora truncata* (Myriaporidae)^1^	—	360.0 × 350.0/—	?

1 In the absence of the data on mature oocytes, measurements of the early and late embryos allowed only approximate calculation of the embryonic enlargement.

The number of oocytes and embryos measured for each species differed depending on specimen availability. Although some abundant species were represented by several colonies, others (often collected from remote areas or deep-water) were represented by only a few small fragments, precluding consideration of egg/embryo size variability. For each species, fragments of three colonies containing mature coelomic oocytes and embryos within brood chambers were sectioned. Length and width measurements (defined as two longest perpendicular diameters) were obtained from 5 to 10 randomly selected mature oocytes, zygotes, and late embryos using a microscope fitted with an eyepiece reticule.

In every species, the embryonic increase during incubation was determined by the volume of the largest oocyte being compared with that of the largest late embryo. In the colonies of *Costaticella solida* and *Watersipora subtorquata*, mature eggs were not found, and the corresponding comparison has been made using smallest early (without cilia) and largest late (ciliated) embryos. Oogenesis mode was possible to recognize using early embryos in these species. In *Pterocella scutella*, *Gregarinidra serrata*, and *Micropora notialis*, only mature oocytes or zygotes and early embryos were found. In *Klugeflustra antarctica*, *Costaticella bicuspis*, and *Myriapora truncata* colonies contained only early embryos. For the latter six species, the data on the embryonic enlargement are considered as preliminary. However, this circumstance did not affect the general picture obtained because the embryonic increase in size considered together with the anatomical and cytological data—multiplication, hypertrophy, cytoplasm content and staining of embryophore cells, difference between the size of the mature oocyte/early embryo and brooding cavity and change in size, shape, and distribution pattern of the yolk granules in the embryonic cells.

## Results

Cheilostome bryozoans possess five reproductive patterns, defined as specific sets of reproductive traits, including mode of oogenesis, site and time of fertilization, presence or absence of the parental care and its mode (viviparity or brooding, matrotrophic or nonmatrotrophic), and larval type (Reed 2011; Ostrovsky et al. 2007). A few species with pattern I produce numerous small microlecithal ( = oligolecithal) eggs that after near/postovulatory coelomic fertilization are freely spawned into the water column and develop into long-living planktotrophic larvae. Four other patterns are characterized by an early intraovarian fertilization and development of short-living nonfeeding larva. The most common is pattern II, involving the production of a few large macrolecithal oocytes, followed by embryonic brooding either in the external skeletal chambers (ovicells) or in the internal brooding sacs. In pattern III, production of a few small microlecithal or mesolecithal oocytes is followed by matrotrophic brooding. In contrast, in pattern IV EEN during incubation is associated with macrolecithal oogenesis. Pattern V, known only in one cheilostome family (Epistomiidae), combines EEN with viviparity (here, intracoelomic incubation). Patterns III–V can be referred to as matrotrophic patterns.

Of the 21 studied species of cheilostome bryozoans, 8 species from 6 families combined microlecithal/mesolecithal oogenesis and placentotrophy, thus exhibiting the reproductive pattern III (Table 1), whereas 13 species from 10 families combined macrolecithal oogenesis and placentotrophy, thus exhibiting the reproductive pattern IV (Table 2). The comparative anatomical and histological data on oogenesis and embryophores will be presented separately for species displaying reproductive patterns III and IV below.

### SPECIES WITH REPRODUCTIVE PATTERN III

In eight cheilostome species possessing reproductive pattern III, placentotrophy was substantial as evidenced by a large or considerable enlargement of the embryo and by strong to modest hypertrophy of the embryophore epithelial cells (Table 1).

**Figure 1:**
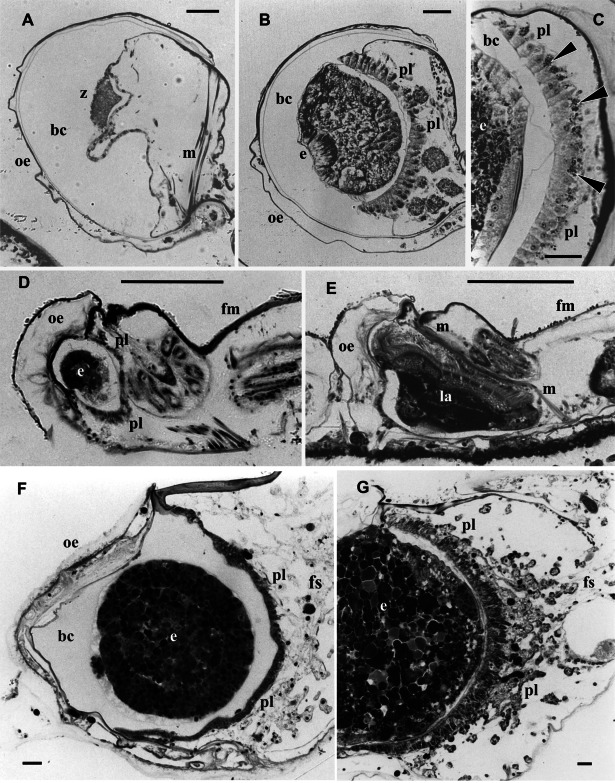
Matrotrophic brooding in *Bugula flabellata* (a,b), *Bugula neritina* (c), *Mollia multijuncta* (d,e), and *Costaticella solida* (f,g). (a) zygote in ovicell (epithelial cells of embryophore are flattened) (sagittal section; zygote shown partially); (b) mid-aged embryo in ovicell with drastically hypertrophied epithelial cells of embryophore; (c) zone of embryo oppsed to embryophore (arrowheads point to dark granules in epithelial cells of placental analogue); (d) early embryo in ovicell with modest hypertrophy of embryophore cells; (e) fully formed larva in ovicell (active nutritional phase is over and cells of placental analogue are flattened); (f) early embryo in ovicell (cells of embryophore in the initial stage of their enlargement); (g) later embryo (placental analogue consists of hypertrophied dark epithelial and lighter funicular cells). bc, brooding cavity; e, embryo; fm, frontal membrane; fs, funicular strands; la, larva; m, muscular bundles; oe, ooecium (protective capsule of the brood chamber); pl, placental analogue (embryophore); z, zygote. Scale bars: a–c, 30 μm; d and e, 100 μm; f and g, 20 μm.

Mature (preovulatory and ovulated) oocytes in these species were micro(oligo)- or mesolecithal (devoid or having few visible yolk granules; Fig. 1a). The minimum size of mature oocytes was 33.6 × 28.8 μm (in *Mollia multijuncta*), whereas the maximum size was 100.0 × 75.0 μm (in *G. serrata*). The maximum embryonic enlargement was found during incubation in *Reciprocus regalis* (257.7-fold) and *Bugula neritina* (310-fold), although in the latter case only submature oocytes and late embryos were available. The minimum enlargement (4.9-fold) was observed in *Cellaria fistulosa*. In all species, the mature oocyte was smaller than the brooding cavity.

In all matrotrophic brooding cheilostomes, EEN is accomplished by the embryophore, a cell complex consisting of the hypertrophied epithelium of the noncalcified wall (or its noncalcified part) of the brood chamber and associated cells of the funicular cords transporting nutrients within a zooid (Fig. 1b–d, g). Providing bidirectional transport of material to and from the embryo (by exo- and endocytosis, correspondingly; Woollacott and Zimmer 1972; Moosbrugger et al. 2012), the embryophore serves as the “maternal part” of the placental analogue. During each brooding episode the embryophore cells are activated and hypertrophied anew, decreasing in size when the brood chamber is emptied.

In five studied species (*Bugula flabellata*, *B*. *neritina*, *Reciprocus regalis*, *Urceolipora nana*, *Pterocella scutella*), when the brood chamber incubated an embryo, the embryophore epithelial cells enlarged drastically, changing their shape from flat to columnar, cubic, or oval (Fig. 1a–c, f, g). Their cytoplasm was intensively or deeply stained (dark) in sections, and sometimes contained numerous dark granules and light vacuoles (*R. regalis*), which was also observed in the funicular cells (*B*. *neritina*; Fig. 1c). Similarly, the epithelial cells in the placental analogue were deeply stained in *Mollia multijuncta* (Fig. 1d) and *Cellaria fistulosa*. Hypertrophy of these cells was modest, however. In the latter species, funicular cells of the embryophore contained dark granules in their cytoplasm and often formed groups.

Colonies of *Gregarinidra serrata* contained only zygotes and very early embryos (Table 1). However, the comparison of the placental analogues in the empty and the incubating brood chambers indicated that embryonic brooding in *G. serrata* was accompanied by proliferation of the epithelial cells of the embryophore. The existence of EEN in this species was also confirmed by the considerable difference between the size of the zygotes and early embryos and that of the brooding cavity.

In *P. scutella*, the well-developed embryophore and more than 33-fold embryonic increase are considered as reliable indicators of EEN, despite the absence of the late embryos in the available material.

### SPECIES WITH REPRODUCTIVE PATTERN IV

Combination of macrolecithal oogenesis and placentotrophy (pattern IV) was recorded in 13 species belonging to 10 families (Table 2). In five species, the oogenesis mode was inferred from observations on zygotes or early embryos in the brood chambers. Embryonic enlargement and hypertrophy of the embryophore cells ranged from negligible to very substantial. Hypertrophy of the embryophore epithelial cells ranged from modest to strong.

The minimum size of the mature macrolecithal (possessing numerous yolk granules) oocyte was found in *Beania bilaminata* (55.2 × 50.4 μm), and the maximum size in *Cribricellina cribraria* (370.0 × 300.0 μm). The greatest embryonic enlargement during incubation was recorded in *B. bilaminata* (468.2-fold), *Celleporella hyalina* (8.8-fold), and “*Calyptotheca” variolosa* (5.57-fold). Because of the absence of the mature oocytes or late embryos in the available material only approximate figures of the embryonic volume increase were obtained for five species: *Klugeflustra antarctica* and *Micropora notialis* (>1.5-fold), *Costaticella bicuspis* (>2.1-fold), *Watersipora subtorquata* (3-fold), and *C. solida* (>4.9-fold; Fig. 1f–g). In the remaining species, the embryonic enlargement calculated varied between 1.27 and 3.39 folds. Noteworthy, in most placental species, the yolk granules in the brooded embryos were larger than in the mature oocytes and had a different shape and, sometimes, color (though they were stained with the same dye). Also, in some instances the large yolk granules, evenly distributed across the cytoplasm in the ripe oocytes, were mainly concentrated in the central area in the embryos.

Similar to the species with reproductive pattern III, degree of embryophore cell hypertrophy and embryonic enlargement during incubation varied greatly. Taking into account these two characters as well as the relationship between the size of the mature oocyte and that of the brooding cavity, the studied species with reproductive pattern IV were classified into four categories.

1. Species with a small embryophore and negligible or little (about 1.5-fold) embryonic enlargement (*K. antarctica, Isosecuriflustra angusta, M. notialis, Figularia figularis*). Mature oocytes and embryos were slightly smaller than the brooding cavity or comparable to it.

*K. antarctica* and *I. angusta* had a small embryophore consisting of enlarged columnar cells with light cytoplasm. In contrast, in *M. notialis* epithelial cells were modestly enlarged and their cytoplasm was intensively stained. In *F. figularis*, a relatively small embryophore consisted of large columnar cell with fine-grained, deeply stained cytoplasm, and associated funicular cells.

(2) Species with the modest hypertrophy of the embryophore cells, but functionally active embryophore and considerable embryonic enlargement (3-fold and more; *Cellaria tenuirostris, C. cribraria, W. subtorquata*). Mature oocytes and early embryos were smaller than the brooding cavity.
(3) Species with a well-developed embryophore of strongly hypertrophied cells and embryonic enlargement from considerable (4.9-fold) to very substantial (468.2-fold). Mature oocytes were distinctly or considerably smaller than the brooding cavity (*B. bilaminata, C. hyalina, “Calyptotheca” variolosa, C. solida, C. bicuspis*).

*Beania bilaminata* is a special case, because it demonstrated the third largest embryonic enlargement ever recorded in cheilostomes (after 500-fold in *Bugula neritina* recorded by Woollacott and Zimmer (1972), and about 1000-fold in viviparous *Epistomia bursaria* recorded by Dyrynda (1948)). In this species, embryophore of large cubic epithelial cells with lightly stained cytoplasm contained numerous dark granules and large light vacuoles. Small dark granules also appeared in the large columnar cells of the placental analogue of “*Calyptotheca” variolosa*. In *C. hyalina*, epithelial and funicular cells of a very large embryophore were intensively stained in sections, and as a result the former were nontransparent (almost black). They were cubic or columnar in shape, forming a tightly packed layer of cells associated with irregular funicular cells (Hughes 1995; Ostrovsky 1981).

Similarly, the placental analogue and funicular system were highly developed in *C. solida* (Fig. 1f–g). The cytoplasm of the hypertrophied epithelial cells contained light vacuoles and small dark granules that were also observed in associated funicular cells of irregular shape. In this species they formed a loose funicular “tissue,” almost completely covering basal parts of epithelial cells and continuous with a dense reticulate network of the funicular cords of the maternal zooid. Both epithelial and funicular cells were intensively stained, but in the former the cytoplasm was much darker. Similar structure of the placental analogue was characteristic of *C. bicuspis*. Despite the absence of late embryos in the available colonies of this species, a well-developed embryophore and size difference between the early embryo and brooding space allowed me to place *Costaticella bicuspis* to the third category.

(4) Species with a well-developed embryophore and supposedly negligible embryonic enlargement (*Myriapora truncata*). Placental analogue consisting of the cubic and columnar epithelial cells with intensively stained cytoplasm and numerous light vacuoles, and associated funicular cells. In this species only early embryos have been found showing that mature oocytes are comparable in size to the brooding cavity.

## Discussion

In addition to contributing new data on oogenesis and the variety of placental analogues, the results of this study suggest the presence in cheilostome bryozoans of the so-called *incipient placentotrophy* (the term coined by Blackburn 2009). Until recently this phenomenon has never been described as such in any invertebrate group. When first recorded in cheilostomes, reproductive pattern IV was considered as an *incipient matrotrophy* (Ostrovsky et al. 2007). However, anatomical analysis has shown that the entire picture of the matrotrophic reproduction in this bryozoan group is much more complex than suggested earlier. The four reproductive categories found among the species with pattern IV allow a detailed reconstruction of hypothetical steps during evolutionary transition from nonmatrotrophic embryonic incubation to the incipient and, further, to substantial placentotrophy accompanied by corresponding changes in oogenesis.

### THE ORIGIN OF PLACENTOTROPHY

In contrast to most other invertebrate phyla, in Bryozoa EEN is a common phenomenon (Levin and Bridges 2005; Ostrovsky et al. 2007; Lidgard et al. 1998). All matrotrophic bryozoans are equipped with temporary structures that, together with the apposed part of the embryo, act as a “simplified placenta-like system” (Woollacott and Zimmer 2010, 1975; see also Supplementary Materials 3). A recently proposed scenario describing the main steps of the advent of placentotrophy in cheilostome bryozoans (Ostrovsky et al. 2007) suggests that the evolution of the new reproductive patterns proceeded as a cascade of events including transitions from the reproductive patterns I to II, from patterns II to IV, and further from patterns IV to III. These transitions involved two corresponding shifts in oogenesis from oligo- to macrolecithal (during transition from patterns I to II) and back (from patterns IV to III). The latter shift could be triggered by acquisition of placentotrophy during incubation, which gradually substituted ovarian vitellogenesis as a major source of the nutrients for embryonic development. An inverse correlation between the degree of the maternal provisioning during oogenesis and matrotrophic gestation is well-known among both invertebrates and vertebrates. For instance, less yolky eggs are known to develop in echinoderm species possessing EEN (Byrne 2008; Wray 1992; Byrne and Cerra 1983; Byrne et al. 1996). Greatly reduced vitelline systems are characteristic of some matrotrophic monogenean flatworms (Cable and Tinsley 1991). Such reduction is considered to be an evolutionary trend in matrotrophic cestodes (Swiderski and Xylander 1992; Korneva 1987 and references therein), and the same trend can be also inferred from the data on egg types in scorpions (Francke 2003) and matrotrophic isopods (Hoese and Janssen 1977). Among vertebrates, some highly placentotrophic squamate reptiles ovulate eggs with a reduced egg content (reviewed in Blackburn 2009). Finally, in mammals evolution of placentation resulted in a shift to microlecithal oogenesis based on the loss of the yolk genes (Rothchild 2007a; Brawand et al. 1985).

Why nutrient transfer during incubation should have evolved in bryozoans is unclear. One possibility is that initially it was relatively unimportant and played no role in the embryonic development. The next step could have arisen as a precocious ovulation and oviposition, as a result of a nonmature egg being transported to the incubation chamber. In this way the role of EEN in provision of resources to the embryo may have gradually changed from supplementary to central. This change is likely to have accompanied a transition from weakly functioning (or small) to active embryophore, and, thus, from incipient to substantial placentotrophy.

The commonly accepted scenario for the evolution of matrotrophy is based on the development of viviparous vertebrates (see Packard et al. 2009b; Blackburn 1989, 2009, 1993, 2005, 2006). In this case, internal fertilization followed by the retention of the eggs are considered as major preconditions. Primitive fetal nutrition was strictly lecithotrophic and was developed further by the addition of small quantities of nutrients from the reproductive tract of the viviparous female. This so-called “incipient matrotrophy” is considered as an initial step toward the evolution of the “specialized” (Wourms 1975) or “substantial matrotrophy” (Blackburn 1989) that was accompanied by a subsequent shift in oogenesis. Examples corresponding to this scenario have been thoroughly studied in squamate reptiles (reviewed in Blackburn 1989) and poeciliid fishes (Reznick et al. 2002; Pollux et al. 2008; Marsh-Matthews et al. 1941, reviewed in Wourms 1975; Marsh-Matthews 1998). When we deal with placenta-like systems, the term “incipient placentotrophy” can be applied (Blackburn 2009, 2005).

As for invertebrates, incipient matrotrophy (and, sometimes, placentotrophy) almost certainly exists among onychophorans (Anderson 1973), scorpions (Farley 1972), and insects (Hagan 1951), although no definitive statements concerning this phenomenon were made.

The results of my research indicate that both incipient and substantial placentotrophy is present among cheilostome bryozoans. Moreover, the finding of different oogenesis modes and degrees of embryonic enlargement and embryophore development in a variety of species (sometimes, from the same genera) allows an insight into the scenario of transition from one of these nutritional modes to the other. Small embryophores in the bryozoan species with large macrolecithal oocytes (*KLugeflustra antarctica*, *Isosecuriflustra angusta*, *Micropora notialis*, and *Figularia figularis*—pattern IV, first category) and a slight/negligible (ca. 1.5-fold) enlargement of the embryo in them suggest, taken together, little nutrient supply (for further discussion see Supplementary Materials 4). If it is absent, then hypertrophy and increase in the number of the embryophore cells together with the change in the staining of their cytoplasm might be explained, for instance, by the active gas exchange and/or removal of waste material from the brood chamber. The most important sign of maternal–fetal physiological relationships here is a recognizable response of the maternal cells to an appearance of a zygote in the brood chamber, which points to the molecular transport. Even if the nutrition is absent, an establishment of such relationships can be the basis for further acquisition of matrotrophy. To note, an occurrence of mother-to-embryo nutrient transfer has been recorded in experiments in a number of poeciliid teleost fishes with large yolky eggs (Marsh-Matthews et al. 1941).

In three species from the second category (pattern IV, *Cellaria tenuirostris*, *Cribricellina cribraria*, and *Watersipora subtorquata*), the embryo becomes noticeably larger (from 3- to 3.39-fold increase) in comparison with mature eggs despite the fact that hypertrophy of the embryophore cells in these species is rather modest. Thus, a degree of the morphological development of the placental analogue is not necessarily directly correlated with its nutritive activity. Elaboration of the placental structures has been known to correlate with a degree of nutritional provisioning during gestation in teleost fishes (Turner 2009) and some scincs (Flemming and Blackburn 2001), but my data show that it is not always the case in cheilostomes.

In the cheilostomes belonging to the third category (pattern IV, *Beania bilaminata*, *Celleporella hyalina*, “*Calyptotheca” variolosa, Costaticella solida*), the embryonic enlargement is substantial or even very substantial and comparable with that in the species with pattern III. Actually, except for the differences in oogenesis mode (macrolecithal vs. oligolecithal), these two reproductive variations are identical, both possessing an embryophore with strongly hypertrophied cells and eggs that are considerably smaller than the brooding cavity.

Based on this information, it might be suggested that a combination of large macrolecithal oocytes comparable in size to that of the brooding cavity, and the small embryonic enlargement provided by a small embryophore (pattern IV, first category) correspond to the earliest stage in the evolution of placentotrophic incubation. Cheilostome bryozoans from the second and the third categories with macrolecithal oocytes of various sizes (but always smaller than the brooding cavity) and different degree of the embryophore development could demonstrate the next step, representing an intermediate stage in the evolution of substantial placentotrophy (see also Moosbrugger et al. 2011). Bryozoan species assigned to these two categories possibly illustrate two different evolutionary trajectories with modest (second) and strong (third) hypertrophy of the cells of the placental analogue, both resulting, however, in a substantial embryonic enlargement (from 3- to 468.2-fold). In general, these species exhibit the entire range of the egg sizes from large (more than 300.0 μm in *C. cribraria*) to tiny (about 50.0 μm in *B. bilaminata*) and embryophore development, thus demonstrating a decrease in the size of macrolecithal oocytes, a corresponding drop in the ovarian activity and, oppositely, an increase in the placental activity. Thus, a shift from the incipient to the substantial matrotrophy/placentotrophy occurred in species with macrolecithal oogenesis. Until now such variation in the maternal provisioning and placental structure has been recorded only in squamate reptiles (Stewart 1999; Blackburn 2009, 1992; Stewart and Thompson 1945) and some teleost fishes (Turner 2009; Wourms 1975; Reznick et al. 2002; Marsh-Matthews et al. 1941).

The final step in this hypothetical transition from patterns IV to III was a change from the production of small macrolecithal to meso- or oligolecithal eggs, supported by a substantial placentotrophy. Among the species with pattern III the maximum embryonic enlargement was recorded in those with the smallest oocytes (*Bugula neritina*, *Reciprocus regalis*, *Mollia multijuncta, Pterocella scutella*; see Table 1), and it is in these species that the difference between the egg size and the brooding cavity size was the most prominent.

Similarly to pattern IV, species with pattern III demonstrate different degrees of embryonic enlargement and embryophore development. There is no clear correlation between these two characters, however, and both species with strong and modest hypertrophy of the cells of the placental analogue demonstrated a big variation in the embryonic enlargement. For instance, species with a modest hypertrophy of the embryophore cells showed a range of this enlargement from 4.9-fold in *C. tenuirostris* to 53.4 in *M. multijuncta*, and so did the species with the large hypertrophy of these cells: from 6.3-fold in *Bugula flabellata* to 310-fold in *B. neritina*. Thus, similarly to pattern IV, two variants of the substantial placentation—with modest and with strong hypertrophy of the placental analogue cells—are detectable among the species producing eggs with small amount of yolk.

The specific case of *Myriapora truncata* (pattern IV, fourth category), which combines large macrolecithal eggs and strong hypertrophy of the embryophore cells, is puzzling. Its zygote occupies the entire cavity of the ovicell so that further embryonic growth would be strongly restricted. This case may be an example of a rapid evolution of a well-developed placental analogue, contrasting with the model of gradual acquisition of the embryophore discussed earlier. Another possible explanation is that in *Myriapora truncata* the embryophore serves exclusively for excretory purposes, removing wastes produced by the large embryo.

### MULTIPLE ORIGINS OF PLACENTOTROPHY IN CHEILOSTOMATA

Matrotrophy and, in particular, placentotrophy are generally regarded as having evolved numerous times in different classes of vertebrates (Wourms 1975; Blackburn et al. 2006; Blackburn 1989, 1993, 2005; Wooding and Burton 2008). Similarly, the distribution of the patterns of sexual reproduction across Bryozoa strongly suggests that placentotrophy evolved independently in all the three bryozoan classes and within both gymnolaemate orders (Ostrovsky 2009; Ostrovsky et al. 2007). Unfortunately, robust phylogenetic framework is still missing for Bryozoa. Published molecular phylogenies are very incomplete (at best species from less than 10% of all described genera were analyzed) and contradictory in many important details (see Tsyganov-Bodounov et al. 1984; Fuchs et al. 1996; Knight et al. 1989; Waeschenbach et al. 2002). As to matrotrophic cheilostomes only few species from the matrotrophic genera *Bugula*, *Beania*, *Watersipora*, *Cellaria*, and species *Bicellariella ciliata* and *C. hyalina* were involved in the molecular analysis, and in all phylogenetic trees published, the distribution of matrotrophic taxa is very patchy. Species of *Bugula* and *Beania* are situated within the same branch in the trees made by Knight et al. (1989), and *Bicellariella ciliata* is placed in the same branch with *Bugula*s in the tree by Tsyganov-Bodounov et al. (1984). Such a placement implies a possibility for a common ancestor with EEN for some lineages within Buguloidea. This suggestion requires, however, more rigorous analysis involving much more taxa from this large superfamily.

One of the major arguments in favor of this suggestion for Cheilostomata is the presence of two, sometimes three patterns of sexual reproduction in the same families and the presence of two patterns in the same genera. In other words, in many instances closely related species can be matrotrophic or nonmatrotrophic, or, if matrotrophic, may have different modes of oogenesis. Species with pattern II (nonmatrotrophic with macrolecithal oogenesis) and pattern IV (matrotrophic with macrolecithal oogenesis) have been recorded in the families Candidae, Cribrilinidae, and Hippothoidae. Patterns II, III (matrotrophic with oligolecithal oogenesis), and IV are known in Bugulidae, Flustridae, Cellariidae, Microporidae, and, apparently, Catenicellidae. Two different patterns has been found in the genera *Gregarinidra* (II and III) and *Isosecuriflustra* (II and IV), and *Cellaria* (III and IV; see also Ostrovsky et al. 2007a). Similar situation has been described in the teleost fishes of the families Poeciliidae and Zenarchopteridae where close relatives “vary either in a presence or absence of matrotrophy or in the degree to which matrotrophy is developed” (Reznick et al. 2002, 2007a, p. 2570). To note, the molecular analysis showed that matrotrophy may have evolved independently not only within these families but also within several different genera (Reznick et al. 2002, 2007a, Pollux et al. 2008; Pires et al. 1977; Meredith et al. 1999).

Noteworthy, among bryozoans various degrees of matrotrophy are reported in the cheilostome genus *Bugula* alone, in which the embryonic enlargement ranges from 6.3- to 500-fold in different species (Woollacott and Zimmer 1972; Dyrynda and King 1982; see also Table 1). This closely reminds a continuum of variation in matrotrophic provisioning recorded in such fish genera as *Poeciliopsis*, *Nomorhamphus*, and *Dermogenys* (see Reznick et al. 2002, 2007a). Also, variation in the degree of EEN has been suggested among populations of the poeciliid fish *Phalloceros caudimaculatus* (see Arias and Reznick 1990).

Intraspecific variation of the oocyte type and larval increase during placentotrophic incubation were detected between distant populations of *Bugula flabellata*. Whereas in Brazil, Corrêa (1991, p. 47) recorded “oligolecithal and homolecithal” eggs of 80.0 μm in diameter (larvae 130.0 μm), in the Irish Sea Dyrynda and King (1983, p. 489) described “telolecithal” (= macrolecithal) eggs of about the same size (77.0 μm) with the larger larval size (150.0 μm) and, thus, increase. My material from New Zealand contained oligolecithal eggs (96.0 × 55.0 μm), and the larvae (160.0×120.0 μm) of an intermediate size between two aforentioned populations. Even if these populations are represented by different (but clearly very closely related) species, presence of two different modes of oogenesis may point to the shift between patterns IV and III within this clade.

### PLAUSIBILITY OF AN ALTERNATIVE SCENARIO

The proposed sequence of events in the evolution of placentotrophy within cheilostome bryozoans may be questioned by making a case for reversibility of matrotrophy. An alternative scenario would then be that EEN originated in a hypothetical early nonmatrotrophic brooder producing relatively small mesolecithal eggs (a hypothetical ancient variant of pattern II resulting in a nonfeeding larva). Further evolution could result in a transition to the incipient and, further, to substantial placentotrophy with corresponding enlargement of both the embryo and the brood chamber (pattern III). Transition to the macrolecithal oogenesis accompanied (or not) by the enlargement of oocytes could lead to pattern IV and ultimately to the suppression of matrotrophy, which could then finally disappear through a shift to pattern II.

However, this alternative scenario is made doubtful by two major arguments: the pattern of EEN distribution among the taxa and the timing of their appearance in the palaeontological record. First of all, though EEN turned out to be more common among Cheilostomata than previously thought (see Supplementary Materials 1), the majority of them are nonplacental. It seems extremely unlikely that matrotrophy evolved in the ancestral brooder, became widespread and then was lost many times in different families. For instance, only one supposedly matrotrophic Recent species (*Crassimarginatella falcata*) is known among Calloporidae, the earliest brooding family known since Albian (Upper Cretaceous) and considered as ancestral for a number of cheilostome lineages, including microporids and cribrilinids (Boardman et al. 2005a; Gordon and Voigt 2000; Gordon 2003). Yet the genus *Crassimarginatella* itself is much younger, having evolved in the Danian (Lower Paleocene). There are more similar examples from the families Microporidae, Cribrilinidae, Poricellariidae, and Hippothoidae.

Second, although it is possible that placental analogues are more widespread than is known in cheilostomes (sexual reproduction has been studied anatomically in species from less than 30% of all families), the scarcity of the genera possessing matrotrophy in the Recent representatives in the studied basal clades, and large gaps between the time of their origins, suggest that placental analogues are unlikely to have evolved early and to have achieved wide distribution in the Cretaceous Cheilostomata. Interestingly, the number of genera with proven or suggested EEN increases considerably in the Tertiary. Eight genera belonging to eight families are known since the Eocene (*Scrupocellaria, Beania, Cellaria, Figularia, Catenicella, Adeonellopsis, Hippopodina, Myriapora*), *Miocene* (*Costaticella, Adeonella, Laminopora, Synnotum, Watersipora, Urceolipora*), Oligocene (*Pterocella*) or have no fossil record, that is evolved relatively recently (*Retiflustra, Isosecuriflustra, Gregarinidra, Klugeflustra, Bugula, Bicellariella, Epistomia, Cribricellina, Reciprocus*). It seems that matrotrophy was becoming more and more common during the cheilostome history, but, again, phylogenetic relationships between taxa including matrotrophic species, together with the distribution of reproductive patterns, point to its independent origins.

As a final remark, in chondrichtian fishes and squamate reptiles, live bearing (and, subsequently, matrotrophy) was much more easily gained than lost (Dulvy and Reynolds 1988; Reynolds et al. 2009; Lee and Shine 2011), and in teleost fishes there is no evidence of such transitions (Goodwin et al. 1990; Mank et al. 2012). Although hypotheses on reversals are actively discussed, most authors tend to consider an acquisition of this novelty as a dominant trend in comparison to its loss (Wourms and Lombardi 1969; Shine and Lee 2003; Blackburn 1999b; Reznick et al. 2002; Pollux et al. 2008, see also Blackburn 2005a, b).

### ADAPTIVE IMPORTANCE OF PLACENTAL ANALOGUES IN CHEILOSTOMATA

If, as shown above, placental analogues indeed evolved so many times in bryozoans, the question arises about the selective importance of this structure.

Existing hypotheses reasonably consider placentation as a byproduct of the evolution of parental care in Cheilostomata. Santagata and Banta (2007b, p. 178) proposed a hypothesis, according to which an initial form of embryonic incubation was “vestibular brooding” further added by placental nutrition in this group. However, vestibular or introvert brooding among cheilostomes is unknown that make the hypothesis of Santagata and Banta (2007b) highly improbable (Ostrovsky 1998; Taylor and McKinney 2000; Ostrovsky et al. 2002). In contrast, Hughes (1995) thought that external brood chambers (ovicells) initially were protective structures, later assuming the function of EEN in some species. The structure of different types of brood chambers, their distribution among cheilostomes as well as fossil evidence all point in favor of this hypothesis.

Dyrynda and Ryland (1981), who described recycling (degeneration followed by regeneration) of a tentacle crown and intestine in the maternal zooid during matrotrophic brooding in *Bugula flabellata*, suggested that the “evolution of embryonic placentation” (p. 255) provided an uninterrupted nutrient supply to the embryo in periods when the feeding organs (polypide) degenerated, thus supporting the maximum larval production in bryozoans with ephemeral colonies. However, in *B. bilaminata*, *W. subtorquata* and in all the studied catenicellids and urceoliporids the polypide never regenerates during placentotrophic incubation. The same is true of species from the family Epistomiidae (pattern V) in which uninterrupted EEN is supported by intracolonial transport of nutrients via funicular cords (Marcus 2005; Dyrynda 1948; Dyrynda and King 1997). Thus, similarly to oogenesis, matrotrophic nutrition occurs independently of the presence or absence of a functioning polypide in the zooid (see Dyrynda and Ryland 1981; Dyrynda and King 1982; Ostrovsky 1981, 2009). This suggests a high degree of colonial integration enabling an inter-zooidal distribution of nutrients to nonfeeding (including, incubating) zooids. Thus, any connection between the advance of placentotrophy and polypide recycling is unlikely.

The role of EEN in accelerating embryogenesis, though possible, is unknown. For instance, in the matrotrophic *C. hyalina* larva is incubated from 12–14 days (Hughes 1995) to 3–4 weeks (Cancino and Hughes 1999). Similarly larval development requires from 10–14 to 30 days in nonmatrotrophic cheilostomes studied (see Silén 1996; Gordon 2002; Nielsen 1987). So, at the moment the data are too scarce to make any conclusions.

Ostrovsky et al. (2007) have suggested that EEN affords simultaneous embryonic development and growth, and thus may accelerate the rate of reproduction in the early part of the process. The first small microlecithal oocyte in the ovary of the species with pattern III should theoretically mature faster than the large macrolecithal egg in nonplacental brooders with pattern II. While the macrolecithal oocyte is maturing, the microlecithal oocyte will be transferred to the ovicell, and the new egg will begin its formation in the ovary immediately after oviposition. In this situation, the speed of embryogenesis would not be important. Of more importance is that the first larvae will be released earlier in matrotrophs because their oogenesis is shorter. For instance, it takes about 6 weeks from the beginning of the egg formation in the ovary until the larval release in nonmatrotrophic cheilostome *Chartella papyracea*, and just 3 weeks in matrotrophic *Bugula flabellata* (Dyrynda and Ryland 1981; Dyrynda and King 1982). Such a strategy (simultaneous embryonic growth and development) would benefit the species with ephemeral colonies that live in seasonal waters, allowing them to occupy free biotopes/niches because of the more rapid production of the first generation of larvae. Indeed, matrotrophy was recorded in the families Bugulidae, Beaniidae, Candidae, Flustridae, Cellariidae, Poricellariidae, Catenicellidae, Urceoliporidae, and Epistomiidae that all possess erect weakly calcified colonies, many of which evidently live just a few months. Brief colony life is also characteristic of hippothoid *C. hyalina* (Eggleston 1983). This suggestion is in accord with the recently discussed data on poeciliid fishes that placentation is associated with an increase in the rate of production of offspring early in life (Pires et al. 1977). However, many cheilostome species with ephemeral colonies have no embryophore (Ostrovsky 1981, 2009; Ostrovsky et al. 2007), and there are some matrotrophic species with long-living, heavily calcified colonies (for instance, species of Adeonidae, and, supposedly, *Myriapora truncata*).

Testing the above suggestion would require a comparison of the rate of reproduction in closely related species with contrasting types of maternal provisioning. Larval production should be faster in the species with higher degree of placentation. In other words, settlement should start earlier because less time will be spent to produce a larva. Also experiments could check in what degree placental nutrition is responsible for the larval size variation (see Supplementary Materials 4 for further discussion). First, variation in oocyte size should be studied to see if it influences the larval size too. If not, then it is namely palcentation that manipulates the larval size. Moreover, in the species with low level of matrotrophy such an influence will be clearly less than in highly matrophic species. Different species from the cheilostome genus *Bugula* would be the ideal model in both cases.

### MATROTROPHY AND EVOLUTION OF SEXUAL POLYMORPHISM IN CHEILOSTOMATA

It seems that evolution of matrotrophy could stimulate the origin of sexual zooidal polymorphism. Harmer (1926) suggested that the change from brooding in the external brood chambers (ovicells) to the incubation in the internal sac “has probably been induced by the supply of an increased amount of nutrient yolk to the embryo” (p. 254). Although the transition from the external to the internal brood chamber in some families was probably associated with the better embryonic protection (see Ostrovsky et al. 2006, 2007, 2009b), matrotrophic incubation inside a voluminous zooid might have resulted in an additional embryonic enlargement. Thus, matrotrophy might stimulate the origin of the sexual polymorphism because many, if not all species from the cheilostome family Adeonidae (presumably entirely placentotrophic) are characterized by a larger orificial size and, in many instances, enlarged brooding zooids. Similarly, EEN might result in the evolution of polyembryony and enlarged gonozooids in the bryzoan class Stenolaemata.

## Conclusions

Although modes of EEN are thoroughly reviewed in vertebrates (Wourms 1975; Wourms et al. 1988; Wourms and Lombardi 1988; Blackburn 1989, 1992, 2005; Blackburn et al. 2006; Wooding and Burton 2008), there has been no attempt to review the topic in invertebrates. Modes of matrotrophy occurring during embryonic incubation include oophagy, adelphophagy, histotrophy, histophagy, and placentotrophy (modified from Blackburn et al. 2006; Blackburn 1992). Chordates possess all these modes, with placentotrophy being most common. It exists in mammals (except monotremes), many squamate reptiles, a relatively large number of bony and cartilaginous fishes, some ascidians and all salps (Wourms 1975; Mukai et al. 2012; Godeaux 1982; Blackburn 2009, 2005; Wooding and Burton 2008). This variety is also represented within the invertebrates, but histotrophy is the most common method. Placentotrophy evolved in Cestoda, Scorpionida, Insecta, Gastropoda, Onychophora, Kamptozoa, and Bryozoa (Hagan 1951; Anderson 1973; Tompa 2000; Nielsen 1991; Reed 2011; Farley 1972; Korneva 1987), but in most of these groups there are only a few placental species. In contrast, scorpions (currently more than 1700 species) are, apparently, all placentotrophic (Farley 1972). The “pseudo-placental viviparity” has been recorded in about 800 species of dipterans, dermatopterans, and psocopterans, and in all the aphids (Hemiptera; about 4000 species; Hagan 1951; Meier et al. 2010; Bermingham and Wilkinson 2000). Considering the enormous overall number of the arthropod species, these figures are perhaps not so surprising. The phylum Bryozoa is much less numerous (about 6000 recent species), but the number of the species with placental analogues is of the same order of magnitude. Placental analogues had evolved in all bryozoan classes, including 87 known species of the freshwater class Phylactolaemata and about 850 species of the recent Stenolaemata (order Cyclostomata). My conservative estimation based on the suggestion that the entire genera *Bugula* and *Watersipora* as well as families Adeonidae and Epistomiidae are matrotrophic gives about 175 species for cheilostomes. Ten species from the gymnolaemate order Ctenostomata also exhibit EEN (reviewed in Ostrovsky et al., 2006). Thus, at least a thousand bryozoan species are placentotrophs, making this phylum the leader among all aquatic invertebrates.

Some variants of placentotrophy in bryozoans are indeed intriguing and merit a full-fledged future research. For instance, in the class Stenolaemata matrotrophy is “two-staged” (in ovary and in membraneous sac) and accompanied by a polyembryony. Species from the class Phylactolaemata develop a tight contact between an embryo and brooding sac wall, suggesting an implantation of the embryonic tissues to the maternal ones reminding the mammalian placenta.

As for Gymnolaemata, the research should target families as Bugulidae and Catenicellidae, which demonstrate different oogenesis modes and possess embryophores with a different degree of the cell hypertrophy. The morphoseries found in these groups suggest a gradual evolution from incipient to substantial placentotrophy within the same clades. A detailed study (both ultrastructural and experimental) of the incipient placentotrophy would be especially important as it is likely to provide an insight into early stages in the evolution of this important novelty.

## Supporting Information

Disclaimer: Supplementary materials have been peer-reviewed but not copyedited.

**Supporting information 1.** Historical overview of the studies on bryozoan placentas.

**Supporting information 2.** Table 1. List of the species studied and collection data.

**Supporting information 3.** Evidence of extraembryonic nutrition in Cheilostomata.

**Supporting information 4.** Critical remarks on methodology.
