# Supplementary material for: FROM INCIPIENT TO SUBSTANTIAL: EVOLUTION OF PLACENTOTROPHY IN A PHYLUM OF AQUATIC COLONIAL INVERTEBRATES
Source: Evolution. 2013 Feb 4;67(5):1368–82. doi: 10.1111/evo.12039 (PMC3698692; doi:10.1111/evo.12039)
Supplement: Supplementary file 1 [file evo0067-1368-SD1.doc]

**Supporting information 1**

***Historical overview***

Extant species from the viviparous order Cyclostomata (class Stenolaemata) and brooding class Phylactolaemata are all matrotrophic. Enlargement of their embryos during incubation has been known for a long time, and supposed placental analogues were described anatomically (Harmer 1893; Braem 1908; Borg 1926; Brien 1953).

In contrast, until recently EEN was considered to be a rare phenomenon among the species of the bryozoan class Gymnolaemata. In fact, there are a number of records in the literature, with examples including the embryonic increase during incubation and the hypertrophied epithelial walls of the brood chambers. Although providing evidence for EEN in the Gymnolaemata these observations appear to have been either forgotten or overlooked (reviewed in Ostrovsky 2008; Ostrovsky et al. 2008). Reid (1845, р. 398) was the first to describe the hypertrophied cellular layer [embryophore] of the distal wall of the ooecial vesicle, closing the opening of the brood chamber (ovicell) in the cheilostome *Bugula flabellata.* He wrote that the “membranous partition [ooecial vesicle] was much thickened, especially at the central part ..., and contained a number of nucleated cells”. The “thickened wall” of the ooecial vesicle during embryonic brooding was also mentioned by Hincks (1861), and was studied in more detail by Vigelius (1886) and Calvet (1900) who used anatomical sections. The embryonic increase in size during incubation has been recorded by several authors (Reid 1845; Hincks 1861, 1873; Nitsche 1869; Vigelius 1886; Calvet 1900) mainly during the study of species of the genera *Bugula* and *Bicellariella* (family Bugulidae). The implications of the observations of these scholars were largely not considered further, although Calvet (1900) noted that the size of the cells of the brooding sac wall, surrounding the embryo in *Cellaria* (Cellariidae) corresponded to a stage in embryonic development.

The hypothesis that an embryo “receives its yolk while in the [brooding] sac” was proposed first by Harmer (1902, р. 301) who compared the sizes of the small oviposed oocyte and of the late embryo, filling half of the zooidal cavity in *Retiflustra schoenaui* (Flustridae). Later, this author (1926, р. 253) mentioned a thickening of the “secretory epithelium [of the brooding sac wall], providing nutriment for the developing embryo” during embryonic incubation, and noted that the late embryo occupied two-thirds of the maternal zooidin in this species. Moreover, he wrote that in the genus *Bugula* “the ovum is small when it first passes into the brood-space. Its increase in size is presumably due to nutriment supplied through the membranous [ooecial] vesicle, which thus acts as a placenta” (p. 203).

Waters (1909, 1912) recorded the internal brooding in eight species from the families Watersiporidae, Adeonidae, Adeonellidae and Beaniidae. In contrast with *Watersipora* sp. (as *Lepralia cucullata*), in which embryos were described and depicted being enveloped by the “thin-walled [internal brooding] sac” (1909, Pl. XV, fig. 4, 1912, p. 495), all other cheilostomes mentioned have been characterized by the “thick-walled sac”. Judging from the Waters’ data (that embryos occupied half or even most of the zooidal cavity in four “adeonid” genera, but the eggs found were from small to moderate size), there is good evidence for extraembryonic nutrition. For instance, this author wrote that in *Beania magellanica* “very large embryo with a thick-walled sac nearly fill[s]… the zooecium” (1912, p. 493). In the subsequent paper Waters (1913) described and depicted hypertrophied epithelium of the brooding sac carrying embryo in *Adeonella lichenoides (as A. platalea*) and *Adeonellopsis crosslandi*. In *Poricellaria ratoniensis* (Poricellariidae) (as *Diplodidymia complicata*) the small egg begins its growth within the small brooding sac, hanging below the zooidal operculum. The embryo was found to be enlarged to such an extent that it filled most of the zooidal cavity, but Waters made no any definite conclusion as to the reason for this. However, he noted the presence of extraembryonic nutrition in the case of *Catenicella elegans* (Catenicellidae) (as *Vittaticella*), since he wrote that there are “several fleshy bands or tubes by which … material for growth is transferred to the ovicell” (1913, p. 484).

Embryonic enlargement and/or placental analogues in *Bugula stolonifera (*as *B. avicularia*), *Celleporella carolinensis* and, possibly, *C. hyalina* (Hippothoidae) (see Ryland, 1979 for discussion) were described and depicted by Marcus (1938). This author wrote that when nourishing an embryo the high cells of the ooecial vesicle produce “albuminous liquid”, i.e.they act as a placenta would. His data on the oocytic and larval size also point to the existence of EEN in *Hippopodina feegensis* (Hippopodinidae), and, supporting data of Waters (1913), in *Catenicella elegans*. In contrast, embryonic increase was not detected in congeneric *C*. *contei*.

Subsequently Marcus (1941, p. 232) reported viviparity (intracoelomic embryonic development) in *Synnotum circinatum* (as *aegyptiacum*) (Epistomiidae). He stated that the embryo “is nourished by the follicle cells which receive alimentary material from other parts of the colony and the maternal brown body, transported by the mesenchymatous tissue-cords”. The late embryo is 50-60 times larger than the mature ovum before cleavage, providing very good evidence for extraembryonic nutrition.

Later, an embryonic increase and a “placenta-like system” were described and /or illustrated in the brooders *Bugula flabellata*, *B. neritina*, *Watersipora cucullata, C. hyaline, C. carolinensis* and *Scrupocellaria ferox* (Candidae) (Corrêa 1948; Mawatari 1952; Woollacott and Zimmer 1972a-b, 1975; Ryland 1979; Dyrynda and Ryland 1982; Dyrynda and King 1983; Hughes 1987; Santagata and Banta 1996; Ostrovsky 1998) and viviparous *Epistomia bursaria* (Epistomiidae) (Dyrynda 1981; Dyrynda and King 1982). Embryonic enlargement was also recorded in *Watersipora arcuata* (Zimmer, pers. com. in Reed, 1991) and *Crassimarginatella falcata* (Calloporidae) (Cook 1985).

Additionally, the existence of EEN in *Bicellariella ciliata* (Bugulidae) was noticed by Ryland (1976), who, using data of Nitsche (1869), compared the sizes of the small oviposed egg and the full-grown larva. Dyrynda and King (1983) also compared the embryonic increase in six matrotrophic species belonging to the genus *Bugula*. Embryos of the different sizes in the ovicells of *Bugula stolonifera* were depicted in the paper of Ryland and Hayward (1992).

Judging from the published descriptions and illustrations (see above), EEN has been recorded in 18 genera belonging to the 14 families of the gymnolaemate order Cheilostomata: in the families Flustridae (*Retiflustra*), Bugulidae (*Bugula*, *Bicellariella*), Beaniidae (*Beania*), Epistomiidae (*Synnotum*, *Epistomia*), Candidae (*Scrupocellaria*), Cellariidae (*Cellaria*), Catenicellidae (*Catenicella*), Hippothoidae (*Celleporella*), Adeonidae (*Adeona*, *Adeonellopsis*), Adeonellidae (*Adeonella*, *Laminopora*), Hippopodinidae (*Hippopodina*), Poricellariidae (*Poricellaria*), Watersiporidae (*Watersipora*) and Calloporidae (*Crassimarginatella*).

A substantial increase in embryo size, sometimes accompanied by changes in the thickness of the brood chamber (introvert), has been reported in eight brooding species of the gymnolaemate order Ctenostomata: in the families Flustrellidridae (*Flustrellidra*), Sundanellidae (*Sundanella*), Nolellidae (*Nolella*), Walkeriidae (*Walkeria*), Mimosellidae (*Bantariella*), and Vesiculariidae (*Zoobotryon*) (Joliet 1877; Hincks 1880; Prouho 1892; Pace 1906; Waters 1914; Braem 1940; Silén 1942, 1944; Banta, 1968). This suggests that they exhibit EEN. Ultrastructural studies of *Z. verticillatum* have also proved a presence of EEN in this species (Ostrovsky and Schwaha, 2011). The structure of the “ectodermic cushion” described in the embryo sac of the “protoctenostome” *Labiostomella gisleni* (Labiostomellidae) (Silén 1944) also suggests that it could be a placental analogue.

My recent study on cheilostome reproductive patterns have revealed matrotrophic characters in species of genera belonging to families Bugulidae (*Bugula*), Beaniidae (*Beania*), Candidae (*Scrupocellaria*), Flustridae (*Gregarinidra*, *Klugeflustra*, *Isosecuriflustra*), Cellariidae (*Cellaria*), Microporidae (*Micropora*), Cribrilinidae (*Figularia*), Catenicellidae (*Paracribricellina*, *Costaticella*, *Pterocella*), Hippothoidae (*Celleporella*), Watersiporidae (*Watersipora*), Myriaporidae (*Myriapora*), Urceoliporidae (*Urceolipora*, *Reciprocus*) andLanceoporidae (‘*Calyptotheca’variolosa*) (Batygina et al. 2006; Ostrovsky 2009; Ostrovsky et al. 2009). One more matrotrophic species belonging to the genus *Mollia* (Microporidae) has been recently recorded (see main text).

As is clearly evident from a detailed analysis of the literature and more recent studies by the author, despite embryonic incubation accompanied by EEN generally being considered as a rare mode of parental care in the class Gymnolaemata, it is in fact much more common. The coverage for this phenomenon is suggested or proven to extend to 39 genera from 26 families. Indirect evidence suggests that there are likely to be more examples, and this is very probable since reproduction of less than 30% of all cheilostome families was studied anatomically. For instance, embryonic increase is seen in the illustration of Kuklinski and Taylor (2006а) in *Harmeria scutulata* (Cryptosulidae).The above-mentioned family-level taxa (those examined directly in the author’s previous studies and those inferred from the literature) are representative of almost half of all gymnolaemate superfamilies. When considered together with the entirely matrotrophic classes Stenolaemata and Phylactolaemata such a wide distribution of EEN within the phylum places Bryozoa among the “most matrotrophic” invertebrate groups such as arthropods and platyhelmints.

**References**

Banta, W. C. 1968. *Mimosella cookae*, new species (Bryozoa, Ctenostomata) with a review of the family Mimosellidae. *Bull. S. Calif. Acad. Sci.* **67**:245-254.

Batygina, T. B., Bragina, E. A., Ereskovsky, A. V., and Ostrovsky, A. N. 2006. Viviparity in plants and animals: invertebrates and lower chordates. St Petersburg State University [In Russian with English summary].

Borg, F. 1926. Studies on Recent cyclostomatous Bryozoa. *Zool. Bidr. Upps.* **10**:181-507.

Braem, F. 1908. Die geschlechtliche Entwicklung von *Fredericella sultana* nebst Beobachtungen über die weitere Lebensgeschichte der Kolonien. *Zoologica* **20**:1-38.

Braem, F. 1940. *Victorella sibogae* Harmer. *Z. Morphol. Okol. Tiere* **36**:267-278.

Brien, P. 1953. Etude sur les Phylactolemates. *Ann. Soc. Roy. Zool. Belg*. **84**:301-440.

Calvet, L. 1900. Contribution à l’histoire naturelle des Bryozoaires Ectoproctes marins. *Trav. Inst. Zool. Univ. Montpel.*, N. S. **8**:1-488.

Cook, P. L. 1985. Bryozoa from Ghana. *Zool. Wetens. Mus. Roy. Afr. Centr. Terv., Belg.* **238**:1-315.

Corrêa, D. D. 1948. A embryologia de *Bugula flabellata* (J. V. Thompson) Bryozoa Ectoprocta. *Bol. Fac. Fil. Sci. Letr. Univ. S. Paulo*, Zoologia **13**:7-71.

Dyrynda, P. E. J. 1981 A preliminary study of patterns of polypide generation-degeneration in marine cheilostome Bryozoa. Pp. 73-81 *in* G. P. Larwood and C. Nielsen eds., *Recent and fossil Bryozoa*. Olsen and Olsen, Fredensborg.

Dyrynda, P. E. J., and P. E. King. 1982. Sexual reproduction in *Epistomia bursaria* (Bryozoa: Cheilostomata), an endozooidal brooder without polypide recycling. *J. Zool. (London)*. **198**:337-352.

Dyrynda, P. E. J., and P. E. King. 1983. Gametogenesis in placental and non-placental ovicellate cheilostome Bryozoa. *J. Zool. (London)*. **200**:471-492.

Dyrynda, P. E. J., and J. S. Ryland. 1982. Reproductive strategies and life histories in the cheilostome marine bryozoans *Chartella papyracea* and *Bugula flabellate*. *Mar. Biol*. **71**:241-256.

Harmer, S. F. 1893. On the occurrence of embryonic fission in cyclostomatous Polyzoa. *Quart. J. Microsc. Sci.* **34**:199-241.

Harmer, S. F. 1902. On the morphology of the Cheilostomata. *Quart. J. Microsc. Sci.* **46**:263-350.

Harmer, S. F. 1926. The Polyzoa of the Siboga Expedition. II. Cheilostomata Anasca. In *Reports of the Siboga Expedition.* Leiden: E. J. Brill, **28b**:181-501.

Hincks, T. 1861. Note on the ovicells of the cheilostomatous Polyzoa. *Quart. J. Microsc. Sci.*, N. S. **1**:278-281.

Hincks, T. 1873. Contributions to history of Polyzoa. *Quart. J. Microsc. Sci.,* N. S. **13**:17-37.

Hincks, T. 1880. A history of the British marine Polyzoa. John Van Voorst, London.

Hughes, D. J. 1987. Gametogenesis and embryonic brooding in the cheilostome bryozoan *Celleporella hyalina.* *J. Zool (London).* **212**:691-711.

Kuklinski, P., and P. D. Taylor. 2006. Unique life history strategy in a successful Arctic bryozoan, *Harmeria scutulata*. *J. Mar. Biol. Assoc. U. K.* **86**:1305-1314.

Joliet, L. 1877. Contributions à l'histoire naturelle des Bryozoaires des côtes de France. *Arch. Zool. Exp. Gen.* **6**:193-304.

Marcus, E. 1938. Bryozoarios marinhos brasileiros, II. *Bol. Fac. Fil. Sci. Letr. Univ. S. Paulo*, Zoologia **2**:1-196.

Marcus, E. 1941. Sobre o desenvolvimento do bryozoario *Synnotum aegyptiacum*. *Arq. Cirurg. Cl. Exper.* **5**:227-234.

Mawatari, S. 1952. On *Watersipora cucullata* (Busk) II. *Misc. Rep. Res. Inst. Nat. Res.* **28**:17-27.

Nitsche, H. 1869. Beobachtungen über die Entwicklungsgeschichte einiger chilostomen Bryozoen. *Z. Wiss. Zool.* **20**:1-13.

Ostrovsky, A. N. 1998. Comparative studies of ovicell anatomy and reproductive patterns in *Cribrilina annulata* and *Celleporella hyalina* (Bryozoa: Cheilostomatida). *Acta Zool.* **79**:287-318*.*

Ostrovsky, A. N. 2008. The parental care in cheilostome bryozoans: a historical review. Pp. 211-245 *in* P. N. Wyse Jackson and M. E. Spencer-Jones eds., *Annals of bryozoology 2: aspects of the history of research on bryozoans*. International Bryozoology Association, Dublin.

Ostrovsky, A. N. 2009. Evolution of the sexual reproduction in the bryozoan order Cheilostomata (Gymnolaemata). St Petersburg State University [In Russian with English summary].

Ostrovsky, A. N., Gordon, D. P., and S. Lidgard. 2009a. Independent evolution of matrotrophy in the major classes of Bryozoa: transitions among reproductive patterns and their ecological background. *Mar. Ecol. Prog. Ser*. **378**:113-124.

Ostrovsky, A. N. and T. Schwaha. 2011. Ultrstructure of the placental analoge in ctenostome bryozoan *Zoobotryon verticillatum* (Delle Chiaje, 1828) (Gymnolaemata). Pp. 254-256 *in* O. V. Zaitseva O.V. and A. A. Petrov eds., *Modern problems of evolutionary morphology of animals. Proceedings of the 2nd All-Russian and international conference dedicated to the 105^th^ anniversary of academician A. V. Ivanov.* Zoological Institute of the Russian Academy of Sciences, St Petersburg [in Russian]

Ostrovsky, A. N, Vavra, N. and J. S. Porter. 2008 Sexual reproduction in gymnolaemate Bryozoa: history and perspectives of the research. Pp. 117-210 in P. N. Wyse Jackson and M. E. Spencer-Jones eds., *Annals of bryozoology 2: aspects of the history of research on bryozoans*. International Bryozoology Association, Dublin.

Pace, R. M. 1906. On the early stages in the development of *Flustrellidra hispida* (Fabricius), and on the existence of a “yolk nucleus” in the egg of this form. *Quart. J. Microsc. Sci.* **50**:435-478.

Prouho, H. 1892. Contribution a l´histoire des bryozoaires. *Arch. Zool. Exp. Gen.* **10**:557-656.

Reed, C. G. 1991. Bryozoa. Pp. 85-245 in A. C. Giese, J. S. Pearse and V. B. Pearse eds., *Reproduction of marine invertebrates. Vol. VI, Echinoderms and lophophorates*. Boxwood Press, Pacific Grove.

Reid, J. 1845. Anatomical and physiological observations on some Zoophytes. *Ann. Mag. Nat. Hist.* **16**:385-400.

Ryland, J. S. 1976. Physiology and ecology of marine bryozoans. Pp. 285-443 *in* F. S. Russell and C. M. Yonge eds., *Advances in marine biology. Vol. 14.* Academic Press, London.

Ryland, J. S. 1979. *Celleporella carolinensis* sp. nov. (Bryozoa Cheilostomata) from the Atlantic coast of America. Pp. 611-619 *in* G. P. Larwood and M. B. Abbott eds., *Advances in bryozoology*. Academic Press, London.

Ryland, J.S. & Hayward, P.J. (1992) Bryozoa from Heron Island, Great Barrier Reef. *Memoirs of Queensland Museum*, 32, 223–301.

Santagata, S., and W. C. Banta. 1996. Origin of brooding and ovicells in cheilostome bryozoans: interpretive morphology of *Scrupocellaria ferox*. *Invert. Biol.* **115**:170-180.

Silén, L. 1942. Origin and development of the cheilo-ctenostomatous stem of Bryozoa. Zool. Bidr. Upps. **22**:1-59.

Silén, L. 1944. The anatomy of *Labiostomella gisleni* Silén (Bryozoa Protocheilostomata). *Kungl. Sven. Vetensk. Handl.*, S. 3, **21**:1-111.

Vigelius, W. J. 1886. Zur Ontogenie der marinen Bryozoen. *Mitt. Zool. Stat. Neap. Rep. Mittelm*. **6**:499-541.

Waters, A. 1909. Reports on marine biology of the Sudanese Red Sea, from collections made by Cyril Crossland, M.A., B.Sc., F.Z.S.; together with collections made in the Red Sea by Dr. R. Hartmeyer. – XII. The Bryozoa. Part I. – Cheilostomata. *J. Linn. Soc., London*, Zoology **31**:123-181.

Waters, A. 1912. A structure in Adeonella (Laminopora) contorta (Michelin) and some other Bryozoa, together with remarks on the Adeonidae. *Ann. Mag. Nat. Hist*., 8 S., **9**:489-500.

Waters, A. 1914. The marine fauna of British East Africa and Zanzibar, from collections made by Cyril Crossland, M.A., B.Sc., F.Z.S., in the years 1901-1902. Bryozoa-Cyclostomata, Ctenostomata and Endoprocta. *Proc. Zool. Soc. London*, Parts **3-4**:831-858.

Woollacott, R. M., and R. L. Zimmer. 1972a. A simplified placenta-like brooding system in *Bugula neritina* (Bryozoa). Pp. 30-31 *in* C. J. Arceneaux ed., *30th Annual Proceedings of the Electron Microscope Society of America*. Claitor’s Publishing Division, Baton Rouge.

Woollacott, R. M., and R. L. Zimmer. 1972b. Origin and structure of the brood chamber in *Bugula neritina* (Bryozoa). *Mar. Biol.* **16**:165-170.

Woollacott, R. M., and R. L. Zimmer. 1975. A simplified placenta-like system for the transport of extraembryonic nutrients during embryogenesis of *Bugula neritina* (Bryozoa). *J. Morph*. **147**:355-378.
