# Supplementary material for: FROM INCIPIENT TO SUBSTANTIAL: EVOLUTION OF PLACENTOTROPHY IN A PHYLUM OF AQUATIC COLONIAL INVERTEBRATES
Source: Evolution. 2013 Feb 4;67(5):1368–82. doi: 10.1111/evo.12039 (PMC3698692; doi:10.1111/evo.12039)
Supplement: Supplementary file 2 [file evo0067-1368-SD2.doc]

**Supporting information 2**

**Table 1.** List of the species studied and collection data.

| № | Species name | area | co-ordinates,  sea | depth | station | date | collecting method,  collector | vessel,  cruise |
| --- | --- | --- | --- | --- | --- | --- | --- | --- |
| 1 | *Gregarinidra serrata* | Spirits Bay,  northern North Island, New Zealand | 34°32΄S  172°79΄E  Fidji Sea | 68m | Z9702,  KAH9901/73 | 28.01.1999 | NIWA/NZOI | r/v “Kaharoa” |
| 2 | *Klugeflustra antarctica* | South Shetland Isl., King-George Isl.,  Ardley Bay | 62°11΄S  58°55΄W | 10 m | St. 3,  sample 10 | 07.12.1990 | boat trawl,  A.F. Pushkin,  A.N. Ostrovsky | 36 Soviet Antarctic Expedition, r/v “Akademician Fedorov”,  5^th^ cruise |
|  |  | South Shetland Isl., King-George Island | 62°17.40΄S  58°31.50΄W | 99m | 190-1 | 05.05.2000 | dredge,  B.I. Sirenko,  I.S. Smirnov | r/v “Polarstern” cruise  ANTXVII/3 |
| 3 | *Isosecuriflustra angusta* | Kapp Norvegia,  Antarctic | 71°11.30΄S  12°15.40΄W | 309-318m | 85-1 | 02.04.2000 | bottom trawl,  B.I. Sirenko,  I.S. Smirnov | r/v “Polarstern”, cruise  ANTXVII/3 |
| 4 | *Bugula flabellata* | Shelly Bay, Wellington Harbour, New Zealand | 41°18΄S  174°47΄E | 5 m |  | July 1997 | boat trawl,  D.P. Gordon |  |
| 5 | *Bugula neritina* | Barbados Isl. | 13°09.14΄N  59°32.50΄W  Caribbean Sea | intertidal |  | 09.05.1972 |  |  |
| 6 | *Beania bilaminata* | Spirits Bay,  Northern North Island,New Zealand | 34°31΄S  172°80΄E  Fiji Sea | 76м | Z9701,  KAH 990/69 | 28.01.2001 | NIWA/NZOI | r/v “Kaharoa” |
| 7 | *Micropora notialis* | Sea of Cosmonauts,  Antarctic | 67°04.90΄S  46°25.69΄E | 230-250m | Station11 | 24.12.1990 | bottom grab,  A.F. Pushkin,  A.N. Ostrovsky | 36 Soviet Antarctic Expedition, r/v “Akademician Fedorov”,  5^th^ cruise |
|  |  | North of Kapp Norvegia,  Antarctic | 71°08.90΄S  13°12.80΄W | 765-840m | Station 138-1 | 11.04.2000 | epibenthic trawl,  B.I. Sirenko,  I.S. Smirnov | r/v “Polarstern”, cruise  ANTXVII/3 |
| 8 | *Mollia multijuncta* | Sormiou Island,  near Marseille, | 43°12.12΄N  05°25.01΄E  Mediterranean | 23 m |  | 17.03.1998  19.03.1998 | SCUBA,  J.-G. Harmelin |  |
| 9 | *Cellaria tenuirostris* | Barrets Reef, entrance to Wellington Harbour,New Zealand | 41°17.07΄S  174°47.29΄E | 10-15 m |  | 05.07.2001 | SCUBA  NIWA |  |
| 10 | *Cellaria fistulosa* | Fetovaia,  Elba Island | 42°43΄N  10°09΄E  Mediterranean | 10-15 m |  | 06.04.1999 | SCUBA,  S.I.Fokin |  |
| 11 | *Figularia figularis* | Grand ConglueIsland,  nearMarseille | 43°10.5΄N  05°24΄E  Mediterranean | 40m |  | 11.10.2001 | SCUBA,  J.-G. Harmelin |  |
| 12 | *Paracribricellina cribraria* | Barrets Reef, entrance to Wellington Harbour, New Zealand | 41°17.07΄S  174°47.29΄E | 10-15 m |  | 05.07.2001 | SCUBA,  NIWA |  |
| 13 | *Costaticella solida* | Barrets Reef, entrance to Wellington Harbour, New Zealand | 41°17.07΄S  174°47.29΄E | 10-15 m |  | 05.07.2001 | SCUBA,  NIWA |  |
| 14 | *Costaticella bicuspis* | Barrets Reef, entrance to Wellington Harbour, New Zealand | 41°17.07΄S  174°47.29΄E | 10-15 m |  | 05.07.2001 | SCUBA,  NIWA |  |
| 15 | *Pterocella scutella* | Barrets Reef, entrance to Wellington Harbour, New Zealand | 41°17.07΄S  174°47.29΄E | 10-15 m |  | 05.07.2001 | SCUBA,  NIWA |  |
| 16 | *Celleporella hyalina* | Matrenin Island, Tchupa Inlet,  Kandalaksha Bay | 66°18.36΄N  33°38.03΄E  White Sea | 3-6 m |  | 02-03.09.1995  11.08.1996 | dredge,  N.N. Shunatova |  |
| 17 | *‘Calyptotheca’ variolosa* | Surrey River,  около Portland,  ю.Австралия | 38°16.8'S  141°41.6'E | 20m |  | 28.11.1981 | SCUBA,  P. Bock,  D.Staples |  |
| 18 | *Watersipora subtorquata* | Wellington Harbour, New Zealand | 41°18΄S  174°47΄E | 1 m |  | 15.12.2001 | D.P. Gordon,  A.N. Ostrovsky |  |
|  |  | Wellington Harbour, New Zealand |  | 1 m |  | November 2007 | M.Carter |  |
| 19 | *Myriapora truncata* | Sormiou Island,  near Marseille | 43°12.12΄N  05°25.01΄E  Mediterranean | 23 m |  | 17.03.1998  19.03.1998 | SCUBA,  J.-G. Harmelin |  |
| 20 | *Urceolipora nana* | off Nowra, New South Wales | 34°55.8'S  151°08.1'E | 429 m | SLOPE 56 | 19.07.1999 | epibenthic trawl  G.C.Poore | r/v ”Franklin” |
| 21 | *Reciprocus regalis* | Spirits Bay,  Northern North Island, New Zealand | 34°32΄S  172°79΄E  Fiji Sea | 68m | Z9702,  KAH 9901/73 | 28.01.1999 | NIWA/NZOI | r/v “Kaharoa” |
