# Supplementary material for: FROM INCIPIENT TO SUBSTANTIAL: EVOLUTION OF PLACENTOTROPHY IN A PHYLUM OF AQUATIC COLONIAL INVERTEBRATES
Source: Evolution. 2013 Feb 4;67(5):1368–82. doi: 10.1111/evo.12039 (PMC3698692; doi:10.1111/evo.12039)
Supplement: Supplementary file 3 [file evo0067-1368-SD3.doc]

##### **Supporting information 3**

##### **Evidence of extraembryonic nutrition in Cheilostomata**

Apart from the embryonic increase in size (which is small in some species studied) and size difference between the brooding cavity and zygote, a presence of EEN (in the others) can be confirmed by the noticeable changes in the cell morphology of embryophore. During incubation these cells undergo significant changes in their properties, increasing in size (figure 1a-b, h-g), showing granules and vacuoles in their cytoplasm (figure 1c) and often changing their coloration. These changes could testify that they not only transport, but also synthesize nutrients accessible for the embryo. The suggested excretory function of the embryophore should also be considered. It is also clear that activation and functioning of the placental analogue is accompanied by proliferation of both epithelial and funicular cells of the embryophore in at least some species (see also Woollacott and Zimmer 1975). The rise of the funicular “tissue” during incubation is especially impressive in *Celleporella hyalina* and catenicellids of the genus *Costaticella*.

Increased physiological activity of the embryophore cells is also supported by cytological and ultrastructural evidence. In *Bugula neritina* Woollacott and Zimmer (1972a-b, 1975) described and illustrated large dark granules which accumulated in the funicular cells adjoining the basal parts of the epithelial cells, and also in the epithelial cells themselves. My observations confirm these data (figure 1c). Moreover, in two other species, which brood their embryos inside internal sacs (*Beania bilaminata* and *Reciprocus regalis*), similar granules were concentrated exclusively in the epithelial cells of the embrophore, namely in their apical (adjoining embryo) parts. Small dark granules were also recorded in these cells in “*Calyptotheca” variolosa* and *Watersipora subtorquata*. In addition to the granules, large light vacuoles were detected in the epithelial cells of embryophore in *Beania bilaminata, Reciprocus regalis* and *Myriapora truncata*.

Woollacott and Zimmer (1975) and Moosbrugger with co-authors (2012) presented ultrastructural evidence for EEN in *Bugula neritina* and *Bicellariella ciliata*. There are numerous microvilli and secretory vesicles in the apical parts of the epithelial cells of the embryophore, whereas embryonic cells form numerous “deep infoldings” indicating the existence of both exo- and endocytosis. Microvilli surrounding the basal parts of the larval cilia is suggestive of active pinocytosis of the fluid of the brooding cavity in *Celleporella hyalina* as was described by Hughes (1987). Interestingly, the cuticle does not appear to be a barrier for the transport of low-molecular substances from the embryophore to the brooding cavity and back (see also Woollacott and Zimmer 1975; Hughes 1987; Moosbrugger *et al.* 2012). Such nutrient transfer through the cuticle of the maternal body wall is otherwise known only in crustaceans (Hoese and Janssen 1989).

Interzooidal transport of the labeled metabolites via funicular cords has been proven experimentally in bryozoans (Best and Thorpe 2002), so it may be reasonable to suggest that nutrient transfer from the maternal zooid to the embryophore is via funicular cords, whereas cells of the embryophore deliver nutrients to the brooding cavity. Transfer of nutrients to the embryophore via funicular cords is also strongly suggested by the observation of degenerating groups of cells with characteristic yellowish-green colouration which would appear to be remnants of the brown body in *Costaticella solida*. These “granules” were found between and inside the funicular cords, as well as in the intercellular spaces and, possibly, cytoplasm of both epithelial and funicular cells of the embryophore. The data supports the suggestion of Ryland (1976) who proposed that the brown body is utilized for the needs of EEN (see also Dyrynda and King 1983). Cytological mechanisms involved in the destruction of the degenerating polypide and transfer of the resulting products have been described extensively (reviewed by Gordon 1977), but the question remains as to how the (parts of the) collapsed cells of the brown body are moved to the embryophore, and if phagocytosis is involved in this process. For instance, Hageman’s (1983) ultrastructural study detected funicular cells phagocytising ovulated oocytes in *Membranipora serrilamella*.

Another important argument is that in most matrotrophic species studied (including those with a small embryonic increase) the yolk granules in the incubated embryos became larger than in the mature eggs and often acquired a different shape and, sometimes, colour after staining with the same dye. In some instances the large yolk granules that normally are evenly distributed across the cytoplasm in the ripe oocytes, were situated in the central part in the embryo.

In 5 species with both reproductive patterns III and IV so-called “nutrient storage cells” were recorded (in *Pterocella scutella*, *Costaticella solida*, *Watersipora subtorquata, Reciprocus regalis* and *Bugula flabellatа*). These enlarged cells (from 8.0 to 28. 0 µm in diameter) situated singly or in groups on the cystid walls and/or funicular cords were described first by Dyrynda and King (1983, p. 487) in the latter species. Their cytoplasm is intensively stained being fine-grained and often containing large vacuoles. The functional importance of these cells is as yet unknown, but since they were found only in matrotrophic species, one could suppose that these cells may be involved in the EEN, possibly storing and releasing additional nutrients.

There is a chance that EEN might also exist in *Scrupocellaria scruposa* (see Ostrovsky et al., 2009a), but additional research will be needed to confirm this. The epithelial cells in the ooecial vesicle of the ovicell in this species were not larger than any other cells of the body wall, but the formation of a more or less complete layer and the intensive staining during embryonic incubation could be a sign of their enhanced physiological activity. Santagata and Banta (1996) discovered EEN in another representative of this genus, reporting the doubling in size of the embryo in *S. ferox* with well-developed embryophore and macrolecithal oocytes (reproductive pattern IV), which agrees well with my data on *S. scruposa* (1.8-fold).

**References**

Best, M. A., and J. P. Thorpe. 2002. Use of radioactive labelled food to assess the role of the funicular system in the transport of metabolites in the cheilostome bryozoan *Membranipora membranacea* (L.). Pp. 29-35 in P.N. Wyse Jackson, C.J. Buttler and M. Spencer-Jones eds., *Bryozoan studies 2001.* A.A. Balkema Publishers, Lisse, Abingdon, Exton, Tokyo.

Dyrynda, P. E. J., and P. E. King. 1983. Gametogenesis in placental and non-placental ovicellate cheilostome Bryozoa. *J. Zool. (London)* **200**:471-492.

Gordon, D. P. 1977. The aging process in bryozoans. Pp. 335-376 *in* R. M. Woollacott and R. L. Zimmer eds., *Biology of bryozoans.* Academic Press, New York, San Francisco, London.

Hageman, G. S. 1983. A fine structural analysis of ovarian morphology, oogenesis, and ovulation in marine bryozoan *Membranipora serrilamella* (Cheilostomata, Anasca). Unpubl. PhD Thesis. University of Southern California, Los Angeles.

Hoese, B., and H. H. Janssen. 1989. Morphological and physiological studies on the marsupium in terrestrial isopods. *Monit. Zool. Ital.*, NS, **4**:153-173.

Hughes, D. J. 1987. Gametogenesis and embryonic brooding in the cheilostome bryozoan *Celleporella hyalina.* *J. Zool (London).* **212**:691-711.

Moosburgger M., Schwaha T., Walzl M.G., Obst M., Ostrovsky A.N. The placental analogue and the pattern of sexual reproduction in the cheilostome bryozoan *Bicellariella ciliata* (Gymnolaemata). *Frontiers in Zoology* 9: 29. doi:10.1186/1742-9994-9-29

Ryland, J. S. 1976. Physiology and ecology of marine bryozoans. Pp. 285-443 *in* F. S. Russell and C. M. Yonge eds., *Advances in marine biology. Vol. 14.* Academic Press, London.

Woollacott, R. M., and R. L. Zimmer. 1971. Attachment and metamorphosis of the cheilo-ctenostome bryozoan *Bugula neritina* (Linne). *J. Morph.* **134**:351-382.

Woollacott, R. M., and R. L. Zimmer. 1972a. A simplified placenta-like brooding system in *Bugula neritina* (Bryozoa). Pp. 30-31 *in* C. J. Arceneaux ed., *30th Annual Proceedings of the Electron Microscope Society of America*. Claitor’s Publishing Division, Baton Rouge.

Woollacott, R. M., and R. L. Zimmer. 1972b. Origin and structure of the brood chamber in *Bugula neritina* (Bryozoa). *Mar. Biol.* **16**:165-170.

Woollacott, R. M., and R. L. Zimmer. 1975. A simplified placenta-like system for the transport of extraembryonic nutrients during embryogenesis of *Bugula neritina* (Bryozoa). *J. Morph*. **147**:355-378.
