# Supplementary material for: FROM INCIPIENT TO SUBSTANTIAL: EVOLUTION OF PLACENTOTROPHY IN A PHYLUM OF AQUATIC COLONIAL INVERTEBRATES
Source: Evolution. 2013 Feb 4;67(5):1368–82. doi: 10.1111/evo.12039 (PMC3698692; doi:10.1111/evo.12039)
Supplement: Supplementary file 4 [file evo0067-1368-SD4.doc]

**Supporting information 4**

***Critical remarks on methodology***

Conclusions on the presence of the incipient matrotrophy can be criticized because of the lack of data on the intraspecific (intracolonial, seasonal, geographic) variation in the embryophore development and larval size that, given the relatively small sample size, in the species with presumed incipient EEN can be responsible for the embryonic “increase”. Working on living material Cancino and Hughes (1988), Wendt (2000), Marshall with co-authors (Marshall et al. 2003; Marshall and Keough 2003, 2004a, 2006, 2008a-c) and Kosman and Pernet (2009) showed that larval size can both vary and be rather stable in different bryozoan species, both within and between populations. It should be added that by chance all of the species studied are placental brooders belonging to the genera *Celleporella*, *Bugula* and *Watersipora*, some with small, others with substantial embryonic increase during incubation. There is no clear understanding why larval size varies in them, however. In *Bugula neritina* larvae increased with the parent colony wet mass (Marshall et al. 2003), at higher colony densities (Marshall and Keough 2008b) and in the colonies after toxicant exposure (Marshall 2008), and diminished in the experimentally halved colonies (Marshall and Keough 2004b), thus being supposedly dependant on the colony state (see also Marshall and Keough 2009). If to suggest that the egg size is stable then the recorded larval size variation reflects variation in the extraembryonic nutrition, i.e. namely placentas is an “instrument” using which a colony controls the larval size. Such functional ‘flexibilty’ of the bryozoan placental analogues may point to their evolutionary past during which their progressive modification might lead to the acquirement of the substantial matrotrophy. In fact, *Bugula neritina* demonstrates the largest larval increase during brooding ever recorded in brooding cheilostomes.

To note, no research on the egg size variation has been ever done in a course of the abovementioned studies, thus it is not known if (and how) this trait might influence the variation in the larval size. Revealing such a connection could considerably add to our understanding of the larval size variability.

In my fixed material the maximal size of both the mature oocytes and late embryos was stable although the sample size was low in comparison with the studies mentioned above, and volume estimation using histological sections is clearly not as precise as methods used by the above authors. It is clear that larger sample sizes are required to increase statistical power of embryonic enlargement analyses. However, the egg and larval size can be rather similar even in distant populations of the same species (Corrêa 1948; Dyrynda and King 1983). Also, multiplication of the embryophore cells, their hypertrophy and cytological change as well as discrepancy in size between the mature egg and brooding chamber and changes in the yolk content in the embryos altogether point to the existing EEN. Even if the embryonic increase is small and the nutrient transfer is negligible, the morphological evidence strongly supports an assumption that some exchange (more than just of gases and water) exists between the embryo and the parent.

Another critical point is a water absorption by embryos as a reason for their enlargement. In studies on vertebrates, measurements of changes in dry mass are currently the main indicator of the EEN whereas volume and wet mass are not considered as reliable criteria (Blackburn 1994). One reason is that developing embryos always increase in wet mass and volume (due to water uptake), regardless of whether matrotrophy is present.

In contrast with vertebrates, the size increase is still widely used as an evidence of matrotrophy in invertebrates and lower chordates. Experiments with radiolabelling and diet manipulation (Toolson 1985; Hoese and Janssen 1989; Frick 1998) as well as ultrastructural studies (Domenici and Gremigni 1977; Cable and Tinsley 1991; Schwartz and Dimock 2001; Korneva 2005) are very rare. Instead many authors recorded and described anatomical changes in both parent and offspring during incubation considering them as an additional evidence of matrotrophy (e.g. Hagan 1951; Mukai et al. 1987; Farley 2001, etc.). The small size of eggs and embryos is a main obstacle for using the dry mass in the studies of matotrophy in most invertebrates, and there is only paper using this method to study EEN in the terrestrial isopod crustaceans (Lawlor 1976). The same concerns bryozoans in which phylum, apart of ultrastructural evidence (see above), the embryonic enlargement during incubation together with accompanied morphological changes in the embryophore and the embryo are currently the main criteria to distinguish a presence of EEN. The water uptake is obviously has a place, but its degree is unknown. It should be admitted, that some embryonic increase in volume was recorded in a number of non-matrotophic cheilostome brooders. In their vast majority it ranged from 1.05 to 1.3-fold, but reached up to 2.5-fold in a few species (Ostrovsky 1998, 2009). Such an increase is comparable with that recorded in the species with presumed incipient matrotrophy (see Table 2), but there was neither developed embryophore nor detectable changes in the embryonic cells that could evidence the nutrient transfer in the former species suggesting the water uptake in them.

**References**

Blackburn, D. G. 1994. Standardized criteria for the recognition of embryonic nutritional patterns in squamate reptiles. *Copeia* **4**:925-935.

Cable, J., and R. C. Tinsley. 1991. Intra-uterine larval development of the polystomatid monogeneans, *Pseudodiplorchis americanus* and *Neodiplorchis scaphiopodis*. *Parasitology* **103**:253-266.

Cancino, J. M., and Hughes, R. N. 1988. The zooidal polymorphism and astogeny of *Celleporella hyalina* (Bryozoa: Cheilostomata). *J. Zool. (London)* **215**:167-181.

Corrêa, D. D. 1948. A embryologia de *Bugula flabellata* (J. V. Thompson) Bryozoa Ectoprocta. *Bol. Fac. Fil. Ci. Letr. Univ. São Paulo, Zool.* **13**:7-71.

Domenici, L., and V. Gremigni. 1977. Fine structure and functional role of the coverings of the eggs in *Mesostoma ehrenbergii* (Focke) (Turbellaria, Neorhabdocoela). *Zoomorphology* **88**:247-257.

Dyrynda, P. E. J., and P. E. King. 1983. Gametogenesis in placental and non-placental ovicellate cheilostome Bryozoa *J. Zool. (London)* **200**:471-492.

Farley, R. D. 2001. Structure, reproduction and development. Pp. 13-77 *in* P. Brownell and G. Polis eds., *Scorpion biology and research.* Oxford University Press, Oxford, New York.

Frick, J. E. 1998. Evidence of matrotrophy in the viviparous holothuroid echinoderm *Synaptula hydriformis*. *Invert. Biol.* **117**: 169-179.

Hagan, H. R. 1951. Embryology of viviparous insects. Ronald Press, New York.

Hoese, B., and H. H. Janssen. 1989. Morphological and physiological studies on the marsupium in terrestrial isopods. *Monit. Zool. Ital.*, NS, **4**:153-173.

Korneva, J. V. 2005. Placental type interactions and evolutionary trends of development of uterus in cestodes. *J. Evol. Biochem. Phys.* **41**:552-560.

Kosman, E. T., and B. Pernet. 2009. Diel variation in the size of larvae of *Bugula neritina* in field populations. *Biol. Bull.* **216**:85-93.

Lawlor, L.R. 1976. Parental investment and offspring fitness in the terrestrial isopod Armadillidium vulgare (Latr.) (Crustacea, Oniscoidea). Evolution 30: 775-785.

Marshall, D. J. 2008. Transgenerational plasticity in the sea: context-dependent maternal effects across the life history. *Ecology* **89**(2):418-427.

Marshall, D. J., Bolton, T. F., and M. J. Keough. 2003. Offspring size affects the post-metamorphic performance of a colonial marine invertebrate. Ecology **84**(12):3131-3137.

Marshall, D. J., and M. J. Keough. 2003. Variation in the dispersal potential of non-feeding larvae: the desperate larva hypothesis and larval size. *Mar. Ecol. Prog. Ser*. **255**:145-153.

Marshall, D. J., and M. J. Keough. 2004a. Variable effects of larval size on post-metamorphic performance in the field. *Mar. Ecol. Prog. Ser*. **279**:73-80.

Marshall, D. J., and M. J. Keough. 2004b. When the going gets rough: effect of maternal size manipulation on larval quality. *Mar. Ecol. Prog. Ser*. 272:301-305.

Marshall, D. J., and M. J. Keough. 2006. Complex life-cycles and offspring provisioning in marine invertebrates. Integ. Comp. Biol. **46**(5):643 – 651.

Marshall, D. J., and M. J. Keough. 2008a. The relationship between offspring size and performance in the sea. Amer. Nat. **171**:214-224.

Marshall, D. J., and M. J. Keough. 2008b. Offspring size plasticity in response to intraspecific competition: and adaptive maternal effect across life-history stages. Amer. Nat. **171(2)**:225-237.

Marshall, D. J., and M. J. Keough. 2008c. The evolutionary ecology of offspring size in marine invertebrates. Adv. Mar. Biol. **53**:1-60.

Marshall, D. J., and M. J. Keough. 2009. Does intraspecific competition affects offspring provisioning. *Ecology* **90**(2): 487-495.

Mukai, H., Saito, Y., and H. Watanabe. 1987. Viviparous development in *Botrylloides* (compound ascidians). *J. Morph.* **193**:263-276.

Ostrovsky, A. N. 1998. Comparative studies of ovicell anatomy and reproductive patterns in *Cribrilina annulata* and *Celleporella hyalina* (Bryozoa: Cheilostomatida). *Acta Zool.* **79**:287-318*.*

Ostrovsky, A. N. 2009. Evolution of the sexual reproduction in the bryozoan order Cheilostomata (Gymnolaemata). St Petersburg State University [In Russian with English summary].

Schwartz, M.L., and R.V. Dimock. 2001. Ultrastructural evidence for nutritional exchange between brooding unionid mussels and their glochidia larvae. *Invert. Biol.* **120**: 227-236.

Toolson, E. C. 1985. Uptake of leucine and water by *Centruroides sculpturatus* (Ewing) embryos (Scorpiones, Buthidae). *J. Arachnol.* **13**:303-310.

Wendt, D. E. 2000. Energetics of larval swimming and metamorphosis in four species of *Bugula* (Bryozoa). *Biol. Bull.* **198**:346-356.
